# Simulated hurricane‐induced changes in light and nutrient regimes change seedling performance in Everglades forest‐dominant species

**DOI:** 10.1002/ece3.8273

**Published:** 2021-12-14

**Authors:** Jeremy L. May, Steven F. Oberbauer

**Affiliations:** ^1^ Florida International University Miami Florida USA

**Keywords:** *Bursera simaruba*, disturbance ecology, forest communities, Hurricane, nutrient manipulation, *Pinus elliottii* var. *densa*, *Quercus virginiana*, *Taxodium distichum*

## Abstract

Wind damage from cyclones can devastate the forest canopy, altering environmental conditions in the understory that affect seedling growth and plant community regeneration. To investigate the impact of hurricane‐induced increases in light and soil nutrients as a result of canopy defoliation, we conducted a two‐way factorial light and nutrient manipulation in a shadehouse experiment. We measured seedling growth of the dominant canopy species in the four Everglades forest communities: pine rocklands (*Pinus elliottii* var *densa*), cypress domes (*Taxodium distichum*), hardwood hammocks, and tree islands (*Quercus virginiana* and *Bursera simaruba*). Light levels were full sun and 50% shade, and nutrient levels coupled with an additional set of individuals that were subjected to a treatment mimicking the sudden effects of canopy opening from hurricane‐induced defoliation and the corresponding nutrient pulse. Seedlings were measured weekly for height growth and photosynthesis, with seedlings being harvested after 16 weeks for biomass, leaf area, and leaf tissue N and ^13^C isotope ratio. Growth rates and biomass accumulation responded more to differences in soil nutrients than differences in light availability, with largest individuals being in the high nutrient treatments. For *B*. *simaruba* and *P*.* elliottii*, the highest photosynthetic rates occurred in the high light, high nutrient treatment, while *T*. *distichum* and *Q*. *virginiana* photosynthetic rates were highest in low light, high nutrient treatment. Tissue biomass allocation patterns remained similar across treatments, except for *Q*. *virginiana*, which altered above‐ and belowground biomass allocation to increase capture of limiting soil and light resources. In response to the hurricane simulation treatment, height growth increased rapidly for *Q*. *virginiana* and *B*. *simaruba*, with nonsignificant increases for the other two species. We show here that ultimately, hurricane‐adapted, tropical species may be more likely to recolonize the forest canopy following a large‐scale hurricane disturbance.

## Graphical abstract

Wind damage associated with cyclones can devastate the forest canopy, strongly altering environmental conditions in the understory that affect seedling growth and canopy regeneration. To investigate the impact of hurricane‐induced increases in light and soil nutrients as a result of canopy defoliation, we conducted a two‐way factorial light and nutrient manipulation in a shadehouse. Ultimately, the ability of a species to recolonize hurricane damaged canopies may shift community dominance to more hurricane‐adapted species.

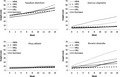

## 1 INTRODUCTION

Cyclones and tropical storms impact many coastal regions all over the world. Hurricanes, cyclones in the Atlantic Ocean basin, are a periodic disturbance producing widespread damage to forest communities (Boose et al., 1994; Imbert, 2018; Stanturf et al., 2007; Zimmerman et al., 1994). Storm damage, and the resulting changes in environmental conditions, has the potential to steer forest successional trajectories (Everham & Brokaw, 1996; Gunderson, 2000; Xi et al., 2019). Understanding the impacts of cyclones and how plant species respond is important to predicting forest structure and community composition.

The magnitude of damage to individual trees within forests often depends to a great extent on canopy position. Canopy‐level trees are likely to be defoliated with snapped branches and trunks or even tipped over from hurricane‐associated winds resulting in the occurrence of canopy gaps (Foster, 1988; Ostertag et al., 2005). Conversely, understory seedlings are often sheltered from most of this damage, although they may be damaged by falling canopy debris (Gilliam et al., 2006; Platt et al., 2000, 2002; Zhang et al., 2008). For trees in the understory, the physical environment can shift markedly following a hurricane, with increases in incoming solar radiation (Battaglia et al., 2001; Carlton & Bazzaz, 1998; Fernandez & Fetcher, 1991), temporary loss of herbivore pressure (Koptur et al., 2002; Luviano et al., 2018), and a pulse of available soil nutrients associated with canopy‐level defoliation (Harmon et al., 1995; Lodge et al., 1991; Ostertag et al., 2003, 2005; Xu et al., 2004).

Increases in available understory light and soil nutrients can be beneficial to seedlings of canopy species, as demonstrated by studies in the treefall gap regeneration literature showing increased growth rates and biomass accumulation (Carrington et al., 2015; Denslow et al., 1998; Grubb et al., 1996; Rodriguez‐Garcia & Bravo, 2013). Changes in available light associated with canopy defoliation can cause both short‐ and long‐term responses in photosynthesis rates and above vs. belowground biomass allocation in seedlings (Cai et al., 2008; Grubb et al., 1996). Differences in phenotypic plasticity between species affect the ability of species to take advantage of dynamic environmental changes via changes in growth, biomass allocation, and photosynthesis (Fetcher et al., 1983, 1985; Monnier et al., 2013; Popma & Bongers, 1988). Disturbance‐adapted species can exhibit substantial plasticity that allows them to reallocate biomass toward the limiting resources that promote success in changing environmental conditions (Schumacher et al., 2009). In contrast, shade‐adapted species may exhibit reductions in growth and photosynthesis in response to sudden increases in light levels (Fetcher et al., 1983; Oberbauer & Strain, 1984).

In addition to shifts in productivity, plant leaf tissues also alter nutrient and stable isotope content in response to varying environmental conditions. Changes in light and soil nutrient conditions affect the uptake and pools of nutrients within plants (Dawson et al., 2002). Tissue nutrient concentrations are affected by a number of environmental factors, including available water, nutrients, and light, with stress contributing to a depletion of nutrient reserves (Dawson et al., 2002; Evans, 2001; Funk et al., 2007; Poorter & Nagel, 2000). Changes in δ^13^C content are negatively correlated with nitrogen and light availability (Guehl et al., 1995; Livingston et al., 1999; van der Sleen et al., 2013), and water stress causing shifts in water use efficiency (Dawson et al., 2002; Warren et al., 2001); however, these could also be indicators of species‐specific intrinsic limitations and ability to respond to environmental stress. Although some field studies have examined tree response to post‐hurricane environments in the Everglades (Platt et al., 2000; Slater et al., 1995; Whelan, 1997), to the best of our knowledge, no studies have experimentally investigated the individual species seedling responses of Everglades canopy dominants to the light and nutrient regimes representative of those before and after a storm.

Our study focuses on four dominant forest communities in the Everglades, which are represented by dominant species from each community. Pine rockland communities are relatively open, fire‐adapted, dry forests dominated by an evergreen conifer, *Pinus elliottii* var. *densa* (South Florida slash pine; Gunderson, 1994; Lockwood et al., 2003; Loope & Dunevitz, 1981). *Pinus elliottii* var. *densa*, South Florida Slash Pine, is a fire‐adapted subspecies of slash pine found in pine rocklands of Central and Southern Florida to the Florida Keys (Critchfield & Little, 1966). Pine rocklands are somewhat resistant to hurricane wind damage, suffering only moderate mortality on canopy individuals (Platt et al., 2000). Cypress domes are long‐hydroperiod (inundated for most of the year), freshwater forests surrounding soil depressions dominated by a deciduous conifer, *Taxodium distichum* var. *nutans* (bald cypress; Gunderson, 1994; Kurz & Wagner, 1953). Hurricanes often cause little major damage or mortality in cypress domes, however, defoliate canopy trees and increase understory light (Noel et al., 1995; Oberbauer et al., 1996). Tree islands are diverse forested patches within the marsh matrix of the Everglades that are situated at higher elevations, causing soils to be drier and better aerated than the surrounding landscape (Bazante et al., 2006; Gunderson, 1994; Loveless, 1959). Similar to tree islands, hardwood hammocks are forests in areas of higher elevation and are characterized by dense growth of temperate and tropical hardwood species (Gunderson, 1994; Olmsted et al., 1980). Regeneration in these two habitats is by both gap‐dependent recruitment and shade‐tolerant replacement. Two species that are common in both hardwood hammock and tree island canopies are a shade‐intolerant tropical deciduous broad‐leaved species, *Bursera simaruba* (gumbo limbo), and a shade‐tolerant, temperate evergreen, broad‐leaved species, *Quercus virginiana* (southern live oak). Both tree islands and hardwood hammocks are impacted by significant damage and mortality resulting from hurricanes (Ruiz et al., 2011).

Here, we examine how seedling growth and allocation of the four Everglade forest‐dominant species respond to conditions representing pre‐ and post‐hurricane light and nutrient availability. The species were chosen to represent dominant species from a range of habitats. Specifically, this study will address the following questions: (1) “How do the seedling growth and allocation responses to altered light and nutrient availability differ among the dominant species from these contrasting communities?”, (2) “How do seedling carbon and nitrogen assimilation patterns compare in response to altered light and nutrient availability?”, and (3) “How do seedling growth rates and biomass allocation change in response to sudden shifts from pre‐hurricane to post‐hurricane environmental conditions?”. We predicted that the species growth and allocation responses would be consistent with tree regeneration patterns and functional traits (e.g., size, tissue nutrient content). Specifically, the light‐demanding *P*. *elliottii* var. *densa* and *B*. *simaruba* are expected to grow best in high light, whereas the shade‐tolerant *Q*. *virginiana* and *T*.* distichum* growth should not respond strongly to light environment. All species are expected to shift tissue allocation toward these limiting resource, light, or soil nutrients, although the degree of reallocation is likely to differ between species. The evergreens *P*. *elliottii* and *Q*. *virginiana* are not anticipated to be as strongly responsive to increased nutrients compared with the deciduous species, *B*. *simaruba* and *T*. *distichum*. Sudden increases in light and nutrients are expected to have an adverse effect on seedlings at first, but seedlings from most species should respond favorably to post‐hurricane conditions with increased growth rates, with the strongest response by the fast‐growing *B*. *simaruba*.

## 2 METHODS

### 2.1 Experimental treatments

We conducted two experiments at the Shadehouse Facility on the campus of Florida International University, Miami, Florida, USA. Seeds or young seedlings sourced from local trees were grown in uniform light and soil conditions to approximately 1–3 months of age; *T. distichum*, *B. simaruba*, and *Q. virginiana* were 1 month old; and *P. elliottii* were 3 months old. Thirty seedlings were randomly assigned to each of the treatment groups and planted individually in 5L, 12 × 30.5 cm tree pots containing a 40:40:20 mix of commercial potting soil:peat moss:sand.

The first experiment used a two‐way factorial design consisting of two light and nutrient levels. The four standard treatments were used to replicate the variety of conditions that seedling naturally grow in and consisted of low nutrient/low light (LNLL), low nutrient/high light (LNHL), high nutrient/low light (HNLL), and high nutrient/high light (HNHL) treatments (*n* = 30 per species). Fertilization rates were 1.5 g phosphorus and 3 g nitrogen per liter of water for high nutrient treatments and distilled water for low nutrient treatments; nutrient levels were determined using a combination of field data and values from previous studies (Rybczyk et al., 1995; Wang et al., 2013). Fertilization of seedlings in the high nutrient treatment group was administered every two weeks. Light treatments were either full sun (~1600–1800 par, high light) or reduced incoming solar radiation using 50% shadecloth (low light). We used a moderate shade level in the low light treatment to reflect light levels in the open canopies of the pineland and cypress communities and to be sure that differences in height growth were a response to resource‐driven growth rather than shade‐driven stem elongation. All plants were well watered twice weekly throughout, and the experiment was conducted over a period of 16 weeks.

In a second experiment, changes in light and nutrients intended to simulate hurricane effects were used to test the plasticity of seedling response. After eight weeks of pre‐hurricane simulation treatment, simulated as low nutrient, low light (LNLL), 30 individuals per species were subjected to a post‐hurricane simulation treatment by placement in high nutrient, high light (HNHL) conditions to simulate an open, post‐hurricane canopy and the storm‐associated litter‐fall nutrient pulse. This transition was accomplished by moving the potted seedlings from the shadehouse into full sun conditions and nutrients added to soil similar to the HNHL treatment described in the first experiment.

### 2.2 Measurements

In both experiments, seedling height and photosynthesis were measured weekly on each individual. Maximum photosynthesis (A_max_, [photosynthetic photon flux density of 1400 µmol m^−2^ s^−1^], flow rate 400 µmol s^−1^, 400 ppm CO_2_) rates were measured using a Li‐6400XT photosynthesis system (LI‐COR Inc., Lincoln, Nebraska, USA) on a subset of 10 randomly selected individuals from each treatment. Plant height was measured weekly and used to calculate absolute growth rate (AGR cm=height at time of measurement) and relative growth rate (RGR %=height at time of measurement x height at start of experiment^−1^). We focused on height growth because occupancy of the canopy was considered the ultimate measure of success. At the conclusion of both experiments, all individuals were harvested and measured for leaf, stem, root biomass, and total leaf area. Biomass was dried at 60°C for 48 h. Leaf tissue of five individuals from each treatment was analyzed for nutrient content (percent nitrogen) and δ^13^C isotopic content using a Thermo Scientific Finnigan Delta‐C Elemental Analyzer‐Infrared Mass Spectrometer (Thermo Fisher Scientific, Waltham, Massachusetts, USA) at the Florida International University Stable Isotope Lab. Carbon isotope ratios were used to test for indication of water and light stress.

### 2.3 Statistical analysis

Seedlings within each treatment were moved every 2 weeks in groupings of five pots during the study in a randomized fashion and compared at the conclusion of the study to confirm that there was no effect of pot placement within the experimental setup. Each species in all the experiments were grouped and modeled independently from one another. Absolute height growth rate (AGR_height_) and relative height growth rates (RGR_height_) were compared using analysis of covariance (ANCOVA) with Tukey's post hoc analysis for the factorial and hurricane simulation treatments. Model intercepts are based on the initial measurement values for each species, and coefficients denote effects of treatment type and magnitude of species response. Comparisons of photosynthesis rates, biomass accumulation, and nutrient content were conducted using two‐factor mixed‐design ANOVAs with a Tukey's post hoc analysis. Comparison of LNLL and post‐hurricane simulation treatments was conducted using a series of t tests. Statistical interactions between light and soil nutrient levels were compared using data from HNHL, LNHL, HNLL, and LNLL treatments. All statistical tests were conducted using the R statistical environment with a significant *p*‐value < .05 (R Core Team, Vienna, Austria).

## 3 RESULTS

### 3.1 Height growth and biomass accumulation

**FIGURE 1:**
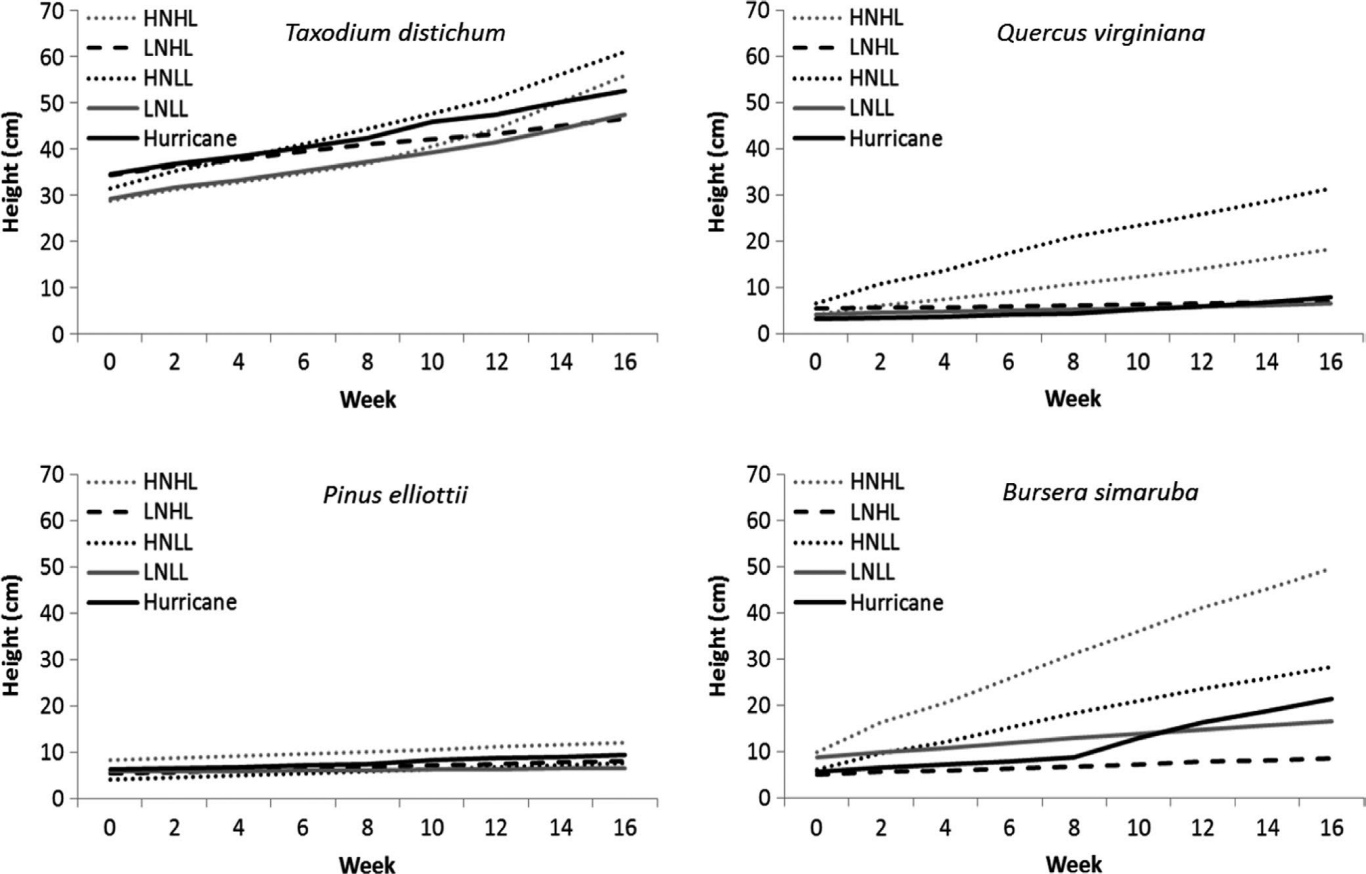
Weekly measurement of height (cm week^−1^) of four common tree species seedlings under altered light and nutrient regimes (HNHL, high nutrient, high light; HNLL, high nutrient, low light; LNHL, low nutrient, high light; LNLL, low nutrient, low light; and hurricane simulation, *n* = 30)

Plant height generally responded more to increases in nutrients than light (Figure 1 and Figure S1). All species showed strong effects of increased nutrients on height growth whereas growth of *P. elliottii* and *T. distichum* was largely unaffected by light availability. *Pinus elliottii* and *B. simaruba* growth rates (AGR_height_)were highest in high nutrient, high light (HNHL) conditions (0.23 and 2.48 cm week^−1^, respectively) and lowest in low nutrient, low light (LNLL) for *Pinus elliottii* (0.05 cm week^−1^, *p *= .002) and low nutrient, high light (LNHL) for *B. simaruba* (0.22 cm week^−1^, *p *< .001). *Taxodium distichum* and *Q. virginiana* AGR_height_ were highest in high nutrient, low light (HNLL) conditions (1.86 and 1.56 cm week^−1^) and lowest in low nutrient, high light (LNHL) (0.77 and 0.10 cm week^−1^, *p* = .003 and *p* < .001, respectively). Across all treatments, *P. elliottii* AGR_height_ were lowest of all the species in the study (0.05 cm week^−1^ LNLL to 0.23 cm week^−1^ HNHL, *p *< .001). *Bursera simaruba* had the highest AGR_height_ of all study species (2.48 cm week^−1^ HNHL); however, across all treatment types, *T. distichum* had the highest average AGR_height_ (0.77 cm week^−1^ LNHL to 1.86 cm week^−1^ HNLL. *p *< .001).

The hurricane simulation treatment increased the growth of the two broad‐leaved species but not the conifers (Figure 1). *Bursera simaruba* showed the largest increase in growth after hurricane simulation treatment (+0.80 cm week^−1^, *p *< .001) and *Q. virginiana* growth rates showed moderate increases (+0.12 cm week^−1^, *p *= .028). For the conifer species, *P. elliottii* and *T. distichum*, growth rates did not significantly change after the after hurricane simulation treatment (+0.03 cm week^−1^, *p *= .791 and −0.29 cm week^−1^, *p *= .361, respectively).

Total dry biomass accumulation differed substantially among species and treatments, with *P. elliottii* and *B. simaruba* accumulating the largest overall biomass (2.3–7.9 g and 0.2–13.0 g, respectively; Table 1 and Table S1). Total biomass was highest for *T. distichum*, *P. elliottii*, and *B. simaruba* in high nutrient, high light (HNHL) treatments (3.9, 7.9, and 13.0 g); however, biomass was highest for *Q. virginiana* in the high nutrient, low light (HNLL) treatment (3.3 g). Across all species, total biomass accumulation was higher in high nutrient (HNHL, HNLL) compared with low nutrient (LNHL, LNLL) treatments. All species, except *Q. virginiana*, had a significant interaction between light and nutrient levels on total biomass. After hurricane simulation, *Taxodium distichum*, *P. elliottii*, and *B. simaruba* significantly increased in overall biomass accumulation (+1.4 g, *p *= .002, +4.8 g *p *< .001, and +0.7 g *p *= .041, respectively; Table 1). In contrast, biomass of *Q. virginiana* did not increase.

**TABLE 1:** Performance of four common tree species seedlings under altered light and nutrient regimes (HNHL, high nutrient, high light; HNLL, high nutrient, low light; LNHL, low nutrient, high light; LNLL, low nutrient, low light; and hurricane simulation, *n* = 30)

	HNHL	LNHL	HNLL	LNLL	LxN	Hurricane
*Taxodium distichum*						
Total biomass (g)	3.9a	2.1b	3.2a	1.9b	*	3.3*
Root % (biomass)	41.9a	42.1a	43.2a	40.5a		42.5
Stem % (biomass)	36.8a	42.0a	35.2a	37.8a		32.9
Leaf % (biomass)	21.3a	15.9a	21.6a	21.7a		24.5
R:S ratio	0.65a	0.75ab	0.82b	0.70a		0.69
*Quercus virginiana*						
Total biomass (g)	0.9a	0.3a	3.3b	0.5a		0.5
Root % (biomass)	50.1a	59.6ab	52.5a	64.4b	*	61.0
Stem % (biomass)	20.5a	18.9a	15.9ab	14.2b		13.1
Leaf % (biomass)	29.3a	21.5b	31.6a	21.4b	*	26.0
R:S ratio	1.0a	1.5ab	1.1a	1.8b	*	1.6*
*Pinus elliottii*						
Total biomass (g)	7.9a	3.0b	6.1ab	2.3b	*	7.1*
Root % (biomass)	42.5a	58.8b	41.2a	42.3a		49.8
Stem % (biomass)	24.4a	23.0a	18.0b	24.6a		24.5
Leaf % (biomass)	33.1a	18.2b	40.8c	33.1a		25.6
R:S ratio	0.70a	1.40b	0.70a	0.70a		1.00*
*Bursera simaruba*						
Total biomass (g)	13.0a	0.2b	4.2c	1.0d	*	1.7
Root % (biomass)	23.1a	28.3a	25.7a	23.4a		20.4
Stem % (biomass)	31.5a	33.5a	30.6a	27.0a		30.3
Leaf % (biomass)	45.4a	38.2a	43.7a	49.6a		49.2
R:S ratio	0.30a	0.40b	0.30a	0.30a		0.30

Note

Letters indicate significance results from Tukey's post hoc analysis on a two‐way analysis of variance between HNHL; HNLL; LNHL; and LNLL. Statistical interaction between light and nutrient levels is denoted as LxN. Asterisks (*) indicate significance results from *t* tests between LNLL and post‐hurricane treatment, as well as LxN. Significance is *p*‐value < .05.

### 3.2 Biomass allocation and leaf area

Root, shoot, and leaf biomass allocation percentages were similar across all treatments for *T. distichum* and *B. simaruba* (Table 1, Supplement 2); however, *P. elliottii* and *Q. virginiana* showed shifts toward greater root allocation when grown in low nutrient (LNHL, LNLL) conditions. *Quercus virginiana* was the only species that showed significant interaction between light and nutrient levels in both percent leaf biomass (*p *< .001) and root:shoot ratio (*p *= .003). Total leaf area was largest for all species in high nutrient (HNHL, HNLL) conditions (Table 2). *Taxodium distichum* and *Q. virginiana* total leaf area was largest under high nutrient, low light (HNLL) conditions (63.8 and 66.1 cm^2^) while *P. elliottii* and *B. simaruba* were largest under high nutrient, high light (HNHL) conditions (76.2 and 398.6 cm^2^). All species showed a significant interaction between light and nutrient levels with regard to leaf area. All species had the highest specific leaf area (SLA) in low nutrient, high light (LNHL) treatments, except *T. distichum* for which SLA was highest in low nutrient, low light (LNLL). Only *T. distichum* showed a SLA significant interaction as a result of light and nutrient conditions (*p *< .001). Root:shoot ratios were highest for *P. elliottii* and *B. simaruba* in low nutrient, high light (LNHL) treatments (1.40 and 0.40) and for *Q. virginiana* in LNLL (1.10) and *T. distichum* in high nutrient, low light (HNLL, 0.82). Across all treatments, *Q. virginiana* had the highest root:shoot ratio (1.00–1.80) and *B. simaruba* had the lowest (0.30–0.40).

**TABLE 2:** Leaf characteristics of four common tree species seedlings under altered light and nutrient regimes (HNHL, high nutrient, high light; HNLL, high nutrient, low light; LNHL, low nutrient, high light; LNLL, low nutrient, low light; and hurricane simulation, *n* = 30)

	HNHL	LNHL	HNLL	LNLL	LxN	Hurricane
*Taxodium distichum*						
Leaf area (cm^2^)	59.4a	29.5b	63.8a	43.7c	*	39.2
SLA (cm^2^ g^−1^)	71.4a	89.5b	92.7b	107.4c	*	48.7*
% *N*	1.86a	1.72ab	1.79a	1.62b	*	2.24*
Leaf *N* area (cm^2^ g^−1^)	0.022a	0.052b	0.026a	0.040c	*	0.028
δ^13^C	−29.21a	−29.0a	−29.4ab	−29.7b		−28.8*
*Quercus virginiana*						
Leaf area (cm^2^)	36.0a	14.2b	66.1c	17.3b	*	20.5
SLA (cm^2^ g^−1^)	130.7a	208.9b	62.4c	172.9d		159.6
% *N*	1.22a	1.22a	1.78b	1.14a		1.25
Leaf *N* area (cm^2^ g^−1^)	0.044a	0.179b	0.017c	0.114d	*	0.097
δ^13^C	−31.1a	−31.3a	−30.4b	−30.3b		−30.9*
*Pinus elliottii*						
Leaf area (cm^2^)	76.2a	25.7bc	45.8b	22.5c	*	70.5*
SLA (cm^2^ g^−1^)	29.0a	46.4b	18.5c	29.2a		39.0*
% *N*	0.73a	0.79a	0.78a	0.65a		0.85
Leaf *N* area (cm^2^ g^−1^)	0.003a	0.014b	0.003a	0.008ab	*	0.005
δ^13^C	−31.0ab	−31.5a	−31.6a	−30.6b		−31.1*
*Bursera simaruba*						
Leaf area (cm^2^)	398.9a	14.3b	62.6c	34.1d	*	55.5*
SLA (cm^2^ g^−1^)	67.6a	207.9b	34.3c	67.7a		66.7
% *N*	2.28ab	2.08ac	2.50b	1.90c	*	2.93*
Leaf *N* area (cm^2^ g^−1^)	0.004a	0.302b	0.014a	0.038c	*	0.035
δ^13^C	−31.0ab	−31.6a	−30.7b	0.00a		−30.1*

Note

Letters indicate significance results from Tukey's post hoc analysis on a two‐way analysis of variance between HNHL; HNLL; LNHL; and LNLL. Statistical interaction between light and nutrient levels is denoted as LxN. Asterisks (*) indicate significance results from *t* tests between LNLL and post‐hurricane treatment, as well as LxN. Significance is *p*‐value < .05.

Biomass allocation was consistent after hurricane simulation treatments (Table 1); however, *Q. virginiana* showed a slight decrease in root allocation (−3.4%) to leaf (+4.6%). Total leaf area also increased between after hurricane simulation treatment in *P. elliottii* and *B. simaruba* (+48.0 and +21.4 cm^2^, both *p *< .001; Table 2). *Taxodium distichum* was the only species that significantly decreased specific leaf area (SLA) after hurricane simulation treatment (−58.7 cm^2^ g^−1^, *p *< .001), while *P. elliottii* was the only species that increased (+9.8cm^2^ g^−1^, *p *= .014). *Quercus virginiana* and *P. elliottii* were the only species that significantly altered root:shoot ratio after hurricane simulation treatment (−0.2, *p *= .037 and +0.3, *p *= .029, respectively).

### 3.3 Photosynthesis

**FIGURE 2:**
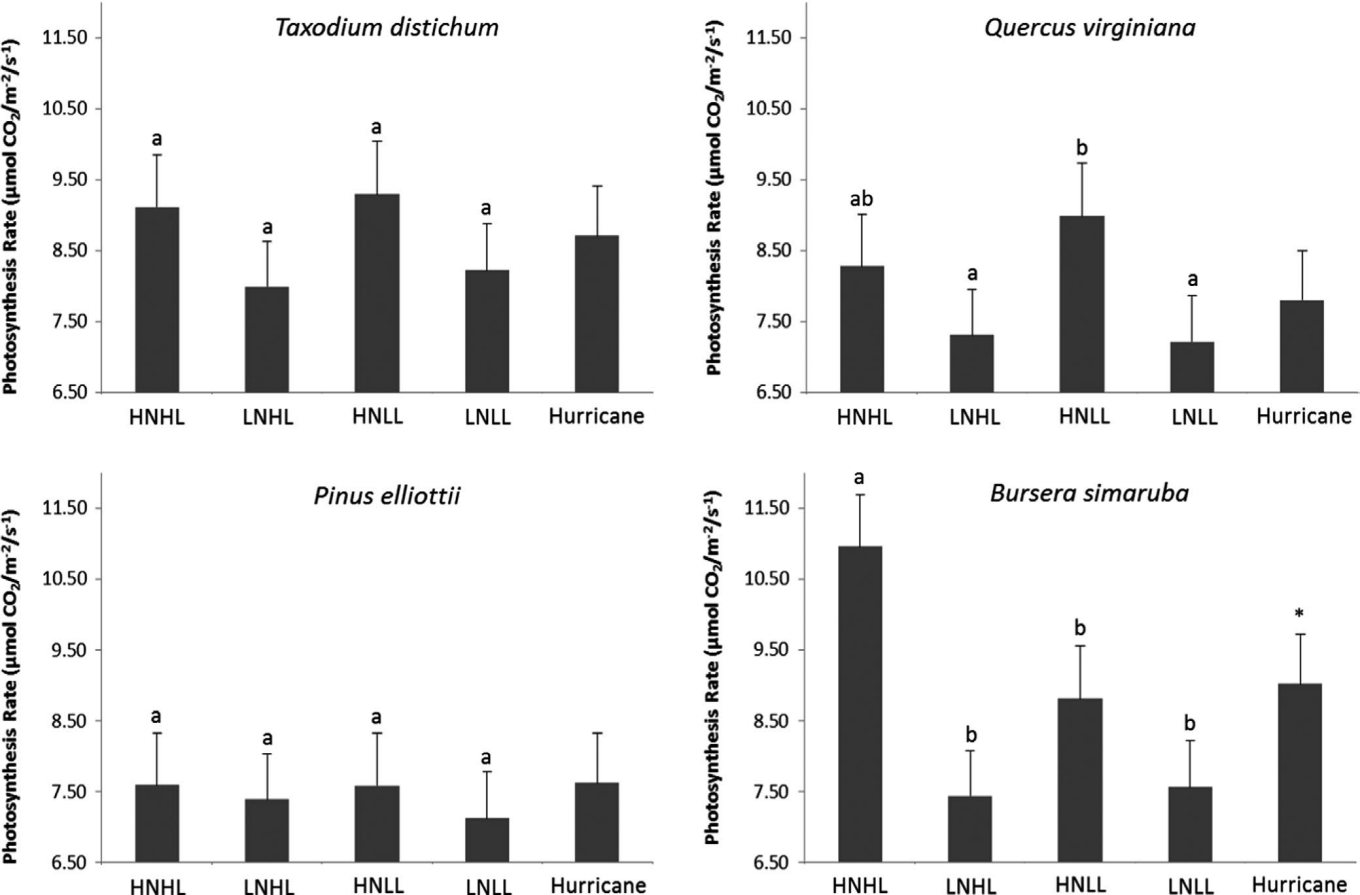
Average weekly photosynthesis rates (µmol CO_2_ m^−2^s^−1^) of four common tree species seedlings under altered light and nutrient regimes (HNHL, high nutrient, high light; HNLL, high nutrient, low light; LNHL, low nutrient, high light; LNLL, low nutrient, low light; and hurricane simulation, *n* = 10). Letters indicate results of Tukey's post hoc analysis, and error bars indicate standard error. Asterisks (*) indicate significance results from *t* tests between LNLL and post‐hurricane treatment. Significance is *p *< .05

Trends in photosynthetic rates across all treatment types were similar to those described for weekly growth rates for all species, with increased nutrient availability having the greatest effect (Figure 2, Supplement 2). Note, while photosynthesis rates were measured weekly, there was little difference over time; therefore, we present study averages only (Figure 2). *Taxodium distichum* and *Q. virginiana* photosynthesis rates were highest in high nutrient, low light (HNLL) treatments (9.30 and 8.99µmol CO_2_ m^−2^s^−1^, respectively) while rates of *P. elliottii* and *B. simaruba* were highest in high nutrient, high light (HNHL) treatments (7.60 and 10.96 µmol CO_2_ m^−2^s^−1^, respectively). For *T. distichum* and *P. elliottii* photosynthesis rates, there were no statistically significant differences among any treatments (*p *= .420 and *p *= .267, respectively). Photosynthetic rate differences after hurricane simulation treatments also were not significantly different for any species, with only *B. simaruba* displaying a noticeable increase post‐hurricane treatment (7.64–9.02 µmol CO_2_ m^−2^s^−1^, *p *= .042).

### 3.4 Leaf N and δ^13^C

Leaf nitrogen content response to differences in light and nutrient availability varied by species (Table 2, Supplement 2). The two broad‐leaved species, *Q. virginiana* and *B. simaruba*, had the highest leaf nitrogen content in high nutrient, low light (HNLL) conditions (1.78% and 2.50%, respectively); however, the conifer species, *T. distichum* (HNHL = 1.86%) and *P. elliottii* (LNHL = 0.79%), had highest values in high light conditions. Leaf nitrogen was lowest for all species in low nutrient, low light (LNLL) conditions. *Pinus elliottii* had the lowest overall leaf nitrogen content (0.65% to 0.79%), and *B. simaruba* had the highest overall nitrogen content (1.90% to 2.50%). *Taxodium distichum* was the only species that showed a significant interaction between light and nutrient levels for nitrogen content (*p *= .041). Leaf nitrogen (N) area was highest in the LNHL treatment for all species. Leaf δ^13^C ratios differed only slightly among treatments within species with ranges less than 1‰ (Table 2). For *T. distichum*, the plants in low nutrient, high light (LNHL) were most enriched while those in low nutrient, low light (LNLL) were most depleted. For *Q. virginiana*, the high light treatments were most depleted and the low light treatments (HNLL, LNLL) most enriched. Leaf isotopic ratios of *P. elliottii* were similar across treatments except for low nutrient, low light (LNLL) that was the most enriched. *Bursera simaruba* δ^13^C ratios were most depleted at low nutrient levels and most enriched at high nutrient levels.

Leaf nitrogen content after hurricane simulation was higher than LNLL treatment in *T. distichum* and *B. simaruba* (+0.58%, *p *= .034 and +1.03%, *p *= .004, respectively; Table 2). No species showed significant differences in leaf N area following the hurricane simulation treatment. All species had significant changes in δ^13^C values after hurricane simulation treatment, although the magnitude and direction of these changes were species specific. After hurricane simulation treatment, *T. distichum* and *B. simaruba* became more enriched in δ^13^C (+0.92‰, *p *= .012 and +1.14‰, *p *= .006, respectively), while *Q. virginiana* and *P. elliottii* became more depleted (−0.54‰, *p *= .023 and −0.47‰, *p *= .028, respectively).

## 4 DISCUSSION

Our study showed variability in the patterns of growth in response to soil nutrient and light conditions across the study species. As expected from previous studies, increased soil nutrient availability resulted in increased growth rates for all species (Grubb et al., 1996; Popma & Bongers, 1988; Rodriguez‐Garcia & Bravo, 2013). Faster growing *B. simaruba* and *T. distichum* were able to alter growth rates in more favorable conditions. The slower growing species, *Q. virginiana* and *P. elliottii*, shifted biomass allocation belowground in response to lower soil nutrient levels. Our results demonstrate that variation in the ability of species to respond to and take advantage of changing environmental conditions may alter the competitive landscape in forest communities following a disturbance.

Responses to changes in light and nutrient availability (i.e., our hurricane simulation) were mixed across the study species; however, the broad‐leaved species showed the most alterations to growth rates and photosynthesis. Abrupt increases in light and nutrient availability resulted in *Q. virginiana* and *B. simaruba* having sustained increased growth rates; however, all species demonstrated a notable short‐term increase in growth after the hurricane simulation treatment. Increases in growth rates resulting from increased availability of light and soil nutrient conditions were consistent with previous findings (Carlton & Bazzaz, 1998; Fernandez & Fetcher, 1991). Only the fastest growing of all the species, *B. simaruba*, increased photosynthesis rates significantly post‐hurricane treatment. Leaf nutrient content and δ^13^C ratios were mixed among species in response to hurricane treatment and inconclusive. The ability to alter growth and be resistant to environmental stresses may help predict which types of species will ultimately be successful in the new conditions following a large‐scale hurricane disturbance. Our results show that faster growing, disturbance‐adapted species, such as *B. simaruba*, becoming more abundant in the community following a disturbance‐induced shift in ecosystem conditions.

### 4.1 Height growth and biomass accumulation

Both light and soil nutrient levels had important effects on height growth and biomass accumulation. Overall, soil nutrient levels had a greater effect on height growth and biomass accumulation compared with the effects of light levels, a finding consistent with previous studies (Denslow et al., 1998; Rodriguez‐Garcia & Bravo, 2013) and somewhat expected given the high light levels. All four species showed strong responses to increased nutrient availability. However, the two broad‐leaved species showed strong, but contrasting, effects of both nutrients and light levels; at high nutrient levels, the shade‐intolerant *B. simaruba* had a significant reduction in height growth with low light level whereas the shade‐tolerant *Q. virginiana* showed an increase in height growth at low light levels. These differences in height growth corresponded to similar patterns in total biomass (Oberbauer & Strain, 1986; Walters et al., 1993). Both broad‐leaved species also showed reduced biomass at in HLLN compared LLLN, though the difference was only significant for *B. simaruba*. The reduction in growth at high light at low nutrient levels does not appear to be an effect of photoinhibition, as photosynthesis rates were not strongly affected by light level at low nutrients (Figure 1), but rather was likely a result of increased allocation to root growth (Table 1). In contrast to the broad‐leaved species, the two conifers both showed weak response to light levels. We had predicted that *T. distichum* would be unresponsive to the difference in light condition, and that appears to also be the case for *P. elliottii*. Our results show that nutrients have a larger influence on growth and biomass allocation compared with light levels, suggesting that soil nutrient limitations may be a driving factor in seedling success.

Height growth rates of the two broad‐leaved species significantly increased as they moved from pre‐hurricane simulation LLLN to post‐hurricane HLHN conditions, with the largest increase in *B. simaruba*. This finding suggests that faster growing species, such as *B. simaruba*, may be able to more effectively take advantage of shifts in their immediate physical environment compared with slower growing, late‐successional species, such as *Q. virginiana*. The ability of *B. simaruba* to quickly increase growth rates under shifts toward more favorable growing conditions suggests that it would be better suited to recolonize the canopy following a disturbance. Both *T. distichum* and *P. elliottii* showed nonsignificant increases in growth rate over the simulated post‐hurricane periods. In the case of *T. distichum*, the absence of increased in growth may have resulted from most individuals losing a portion their leaves when moved from low light to high light conditions. *Pinus elliottii* growth increased >45%, but the effect was not significant as a result of the small absolute changes in height but may also be a result of seedlings having not progressed to the stage at which they increase in height growth quickly.

### 4.2 Biomass allocation

Despite increases in total biomass accumulation with increased resource availability, *T. distichum* and *B. simaruba* showed little difference in biomass allocation among tissues whereas *Pinus elliotti* and *Q. virginiana* showed the largest variation in biomass allocation, similar to previous findings (Jose et al., 2003; Tognetti & Johnson, 1999). *Pinus elliotti* shifted allocation most noticeably from leaves to roots under LNHL conditions, while *Q. virginiana* shifted allocation from roots to leaves in LNLL conditions. Shifts in these two slow‐growing species suggest a plasticity of biomass allocation, allowing them to survive under stressful conditions consistent with growing under canopies and foraging for the most limiting nutrient in their immediate environment (Funk et al., 2007; Poorter & Nagel, 2000).

Biomass allocation in response to the simulated hurricane treatment showed that all of the four species increased in size, but only *Q. virginiana* showed a reallocation of biomass from root tissue to leaves. This ability to reallocate resources as environmental conditions change is beneficial in a species that is slow growing and may be impacted by multiple hurricanes throughout it life. All of the species except *T. distichum* showed an increase in leaf area after simulated hurricane treatment. Loss of leaf area in *T. distichum* was a result of previously mentioned leaf drop at the transition from pre‐ to post‐hurricane simulation, and plants were not able to recover their leaf area over the remainder of the study.

Simulated post‐hurricane conditions and the responses of species to those conditions demonstrate the ability of understory seedlings to regenerate following a storm. In our experiment, individuals of *T. distichum* suffered varying degrees of leaf loss when environmental conditions abruptly changed and did not alter its growth rates in response to newly available resources. However, this response may not affect *T. distichum's* ability to regenerate the canopy of cypress domes because the long hydroperiods, spending much of the year inundated, associated with these habitats suppress the recruitment of other species (Visser & Sasser, 1995). *Pinus elliottii* had the slowest height growth of all the study species; however, after hurricane simulation treatment, it had an increase in leaf area, height growth rate, and, ultimately, total biomass, suggesting an ability to take advantage of changes in resource availability. *Bursera simaruba* was the fastest growing of all the species in optimal conditions and was quickly able to take advantage of the new conditions (post‐hurricane) by increasing its growth rates and leaf area. *Quercus virginiana* showed only a slight increase in growth rate and biomass accumulation in the post‐hurricane simulation this species, however, showed the largest degree of biomass plasticity in allocation allowing it to adjust for changing conditions (Fernandez & Fetcher, 1991; Popma & Bongers, 1988).

### 4.3 Assimilation of C and N

Both conifer species showed no overall change in photosynthetic rates in any treatment, although *T. distichum* had slightly lower mean *A*_max_ in low N conditions. This result, especially in the case of *P. elliottii*, suggests physiological tolerance of low resource levels needed to sustain levels of growth. The two broad‐leaved species (*Q. virginiana* and *B. simaruba*) had the highest photosynthetic rates in nutrient‐rich conditions (HNLL and HNHL, respectively), suggesting that these two species were nutrient limited in the low nutrient treatments. The photosynthetic rates of the two broad‐leaved species also differed in response to the light conditions, similar to previous findings (Fetcher et al., 1987). The fast‐growing, disturbance‐adapted *B. simaruba* had the highest photosynthetic rates under high light conditions while highest rates for the slower growing, late‐successional species *Q. virginiana* were under low light conditions. This difference between the two broad‐leaved species is likely a result of adaptations to seedlings germinating in open (*B. simaruba*) or closed (*Q. virginiana*) canopies. Both *B. simaruba* and *Q. virginiana* had the lowest growth rates in conditions of LNHL, suggesting nutrient availability plays a vital role in how these two species compensate for higher levels of incoming solar radiation and water loss (Denslow et al., 1998; Fetcher et al., 1983).

For each species, treatments with the highest growth rates were generally different from the treatments with the highest nitrogen content, with the exception of *Q. virginiana*. This finding is consistent with those of previous studies in which leaf nitrogen content and growth were not correlated (Funk et al., 2007). Faster growing species may dilute nitrogen content in the plant as biomass is accumulated compared with slower growing species. Alternatively, nitrogen availability may have been above the limiting resource threshold and therefore was not correlated with changes in growth rate.

Carbon isotopic (δ^13^C) enrichment was highest in low light treatments, except *T. distichum*, suggesting that water stress was not a major factor for most of the plants in the study (Warren et al., 2001). δ^13^C was enriched when faster growing individuals (*T. distichum* and *B. simaruba*) were transferred from pre‐ to post‐hurricane simulation conditions, which may have resulted from higher photosynthesis rates. This was not the case in individuals of the slower growing species (*Q. virginiana* and *P. elliottii*), which became more depleted in δ^13^C post‐hurricane simulation. This result may be explained by more conservative growth rates being associated with higher water use efficiency. Increases in incoming solar radiation in the post‐hurricane simulation may play a role in the stomatal conductance through increasing transpiration rates and resulting water use efficiency of these faster growing species (*T. distichum* and *B. simaruba*), resulting in δ^13^C enrichment (Dawson et al., 2002; Guehl et al., 1995).

### 4.4 Responses and life‐history traits

These differences in the height and biomass responses reflect the life‐history traits of the four study species. Overall, each of the study species responded to varying resource availability in different ways that demonstrate differences in adaptive traits. Seedlings of *Q. virginiana and P. elliottii* are slow‐growing species that allocate caloric surplus into storage rather than growth. The *P. elliottii* in this size class are in the “grass” stage, when they store large amounts of photosynthate but have low height growth to keep the meristem low to the ground to survive frequent fires with early growth devoted to deep taproots (Lohrey & Kossuth, 1990). *Quercus virginiana* have large seeds that can germinate in shade generating seedling banks in the understory. We show here that the faster growing, tropical species *B. simaruba* grow best in conditions of high light and soil nutrient content and also is the study species that is most capable of taking advantage of rapidly changing environmental conditions after a hurricane. Differences in seedling growth strategies and ability to take advantage of changing conditions are likely to be good predictors of new canopy recruitment following hurricane disturbance.

Our study shows that slower growing species were less likely to alter growth rates and instead shift biomass allocations. These allocation shifts may aid in allowing them to possibly survive disturbance‐induced environmental shifts although not provide the increases in height growth needed to recolonize a newly opened canopy. In contrast, the faster growing study species, *T. distichum* and *B. simaruba*, demonstrated less change in biomass allocations and increased their growth rates in more favorable conditions, ultimately aiding them in recolonizing canopy gaps. The regenerative niche of *T. distichum* is toward growing quickly before its habitat becomes inundated as to stay above the water level and conspecifics are its primary competition in this habitat. Species‐specific traits and resulting resource utilization habits have the potential to alter competitiveness in response to changing conditions. Under climate conditions where storm damage becomes more frequent, species‐specific abilities to adapt to changing environmental conditions may favor disturbance‐adapted, faster growing species and ultimately lead to long‐term shifts in the community structure and diversity.

In conclusion, nutrient levels had a larger impact than light levels on growth rates and biomass accumulation in this study. The magnitudes of treatment impacts varied considerably and were often species specific. Faster growing species were able to more readily take advantage of favorable conditions through increased growth rates and biomass accumulation, while slower growing species shifted tissue biomass allocation to cope with varying conditions. Broad‐leaved species were able to compensate for sudden changes in light and nutrient availability associated with a simulated hurricane treatment through increased growth rates and biomass accumulation, while coniferous species did not. Responses of these target species to shifts in available light and nutrient may shed light on how other species in the system with similar characteristics may also respond. Variations in species‐specific responses to different environmental conditions may explain how hurricane disturbances can alter the trajectories of community succession in forest systems favoring fast‐growing, tropical species.

## CONFLICT OF INTEREST

None declared.

## AUTHOR CONTRIBUTION

**Jeremy Lee May:** Conceptualization (equal); Data curation (lead); Methodology (equal); Project administration (lead); Writing‐review & editing (lead). **Steven Oberbauer:** Conceptualization (equal); Data curation (supporting); Methodology (equal); Project administration (supporting); Writing‐review & editing (supporting).

### OPEN RESEARCH BADGES

This article has earned an Open Data, for making publicly available the digitally‐shareable data necessary to reproduce the reported results. The data is available at: https://doi.org/10.5061/dryad.c59zw3r89.

## Supporting information

Supplementary Material

## Data Availability

Our data will be publicly accessible and archived on the Dryad database website https://doi.org/10.5061/dryad.c59zw3r89 or through communication with the corresponding author.

## References

[ece38273-bib-0001] Battaglia, L. L. , Fore, S. A. , & Sharitz, R. R. (2001). Seedling emergence, survival and size in relation to light and water availability in two bottomland hardwood species. Journal of Ecology, 88(6), 1041–1050. 10.1046/j.1365-2745.2000.00518.x

[ece38273-bib-0002] Bazante, J. , Jacobi, G. , Solo‐Gabriele, H. M. , Reed, D. , Mitchell‐Bruker, S. , Childers, D. L. , Leonard, L. , & Ross, M. (2006). Hydrologic measurements and implications for tree island formation within Everglades National Park. Journal of Hydrology, 329(3), 606–619. 10.1016/j.jhydrol.2006.03.011

[ece38273-bib-0003] Boose, E. R. , Foster, D. R. , & Fluet, M. (1994). Hurricane impacts to tropical and temperate forest landscapes. Ecological Monographs, 64, 369–400.

[ece38273-bib-0004] Cai, Z.‐Q. , Poorter, L. , Han, Q. , & Bongers, F. (2008). Effects of light and nutrients on seedlings of tropical *Bauhinia* lianas and trees. Tree Physiology, 28, 1277–1285. 18519259 10.1093/treephys/28.8.1277

[ece38273-bib-0005] Carlton, G. C. , & Bazzaz, F. A. (1998). Resource congruence and forest regeneration following an experimental hurricane blowdown. Ecology, 79, 1305–1319.

[ece38273-bib-0006] Carrington, M. E. , Ross, M. S. , & Basit, A. F. (2015). Posthurricane seedling structure in a multi‐aged tropical dry forest: Implications for community succession. Biotropica, 47(5), 536–541.

[ece38273-bib-0007] Critchfield, W. B. , & Little, E. L. (1966). Geographic distribution of the pines of the world. US Department of Agriculture, Forest Service.

[ece38273-bib-0008] Dawson, T. E. , Mambelli, S. , Plamboeck, A. H. , Templer, P. H. , & Tu, K. P. (2002). Stable isotopes in plant ecology. Annual Review of Ecological Systems, 33, 507–559.

[ece38273-bib-0009] Denslow, J. S. , Ellison, A. M. , & Sanford, R. E. (1998). Treefall gap size effects on above‐and below‐ground processes in a tropical wet forest. Journal of Ecology, 86(4), 597–609. 10.1046/j.1365-2745.1998.00295.x

[ece38273-bib-0010] Evans, R. D. (2001). Physiological mechanisms influencing plant nitrogen isotope composition. TRENDS in Plant Science, 6(3), 121–126. 10.1016/S1360-1385(01)01889-1 11239611

[ece38273-bib-0011] Everham, E. M. , & Brokaw, N. V. L. (1996). Forest damage and recovery from catastrophic wind. Botanical Review, 62(2), 113–185. 10.1007/BF02857920

[ece38273-bib-0012] Fernandez, D. S. , & Fetcher, N. (1991). Changes in light availability following hurricane Hugo in a subtropical montane forest in Puerto Rico. Biotropica, 23(4a), 393–399.

[ece38273-bib-0013] Fetcher, N. , Oberbauer, S. F. , Rojas, G. , & Strain, B. R. (1987). Efectos del regimen de luz sobre la fotosintesis y el crecimiento en plantulas de arboles de un bosque lluvioso tropical de Costa Rica. Revista Biologia Tropical, 35(Suppl), 97–110.

[ece38273-bib-0014] Fetcher, N. , Oberbauer, S. F. , & Strain, B. R. (1985). Vegetation effects on microclimate in lowland tropical forest in Costa Rica. International Journal of Biometeorology, 29(2), 145–155. 10.1007/BF02189035

[ece38273-bib-0015] Fetcher, N. , Strain, B. R. , & Oberbauer, S. F. (1983). Effects of light regime on the growth, leaf morphology, and water relations of seedlings of two species of tropical trees. Oecologia, 58(3), 314–319. 10.1007/BF00385229 28310328

[ece38273-bib-0016] Foster, D. R. (1988). Species and stand response to catastrophic wind in central New England, USA. The Journal of Ecology, 135–151. 10.2307/2260458

[ece38273-bib-0017] Funk, J. L. , Jones, C. G. , & Lerdau, M. T. (2007). Leaf‐ and shoot‐level plasticity in response to different nutrient and water availabilities. Tree Physiology, 27, 1731–1739. 17938104 10.1093/treephys/27.12.1731

[ece38273-bib-0018] Gilliam, F. S. , Platt, W. J. , & Peet, R. K. (2006). Natural disturbances and the physiognomy of pine savannas: A phenomenological model. Applied Vegetation Science, 9(1), 83–96. 10.1111/j.1654-109X.2006.tb00658.x

[ece38273-bib-0019] Grubb, P. J. , Lee, W. G. , Kollman, J. , & Wilson, J. B. (1996). Interaction of irradiance and soil nutrient supply on growth of seedlings of ten European tall‐shrub species and *Fagus sylvatica* . Journal of Ecology, 84, 827–840.

[ece38273-bib-0020] Guehl, J.‐M. , Fort, C. , & Ferhi, A. (1995). Differential response of leaf conductance, carbon isotope discrimination and water‐use efficiency to nitrogen deficiency in maritime pine and pedunculate oak plants. New Phytologist, 131, 149–157.

[ece38273-bib-0021] Gunderson, L. H. (1994). Vegetation of the Everglades: Determinants of Community Composition. In S. M. Davis & J. C. Ogden (Eds.), Everglades: The Ecosystem and Its Restoration (pp. 199–215). CRC Press.

[ece38273-bib-0022] Gunderson, L. H. (2000). Ecological resilience: In theory and application. Annual Review of Ecology and Systematics, 31, 425–439.

[ece38273-bib-0023] Harmon, M. E. , Whigham, D. F. , Sexton, J. , & Olmsted, I. (1995). Decomposition and mass of woody detritus in the tropical dry forests of the northeastern Yucatan Peninsula, Mexico. Biotropica, 27(3), 305–316.

[ece38273-bib-0024] Imbert, D. (2018). Hurricane disturbance and forest dynamics in east Caribbean mangroves. Ecosphere, 9(7), e02231. 10.1002/ecs2.2231

[ece38273-bib-0025] Jose, S. , Merritt, S. , & Ramsey, C. L. (2003). Growth, nutrition, photosynthesis and transpiration responses of longleaf pine seedlings to light, water and nitrogen. Forest Ecology and Management, 180(1–3), 335–344.

[ece38273-bib-0026] Koptur, S. , Rodriguez, M. C. , Oberbauer, S. F. , Weekley, C. , & Herndon, A. (2002). Herbivore‐free time? Damage to new leaves of woody plants after Hurricane Andrew. Biotropica, 34(4), 547–554.

[ece38273-bib-0027] Kurz, H. , & Wagner, K. A. (1953). Factors in cypress dome development. Ecology, 34(1), 157–164. 10.2307/1930315

[ece38273-bib-0028] Livingston, N. J. , Guy, R. D. , Sun, Z. J. , & Ethier, G. J. (1999). The effects of nitrogen stress on the stable carbon isotope composition, productivity and water use efficiency of white spruce (*Picea glauca*(Moench) Voss) seedlings. Plant, Cell and Environment, 22, 281–289.

[ece38273-bib-0029] Lockwood, J. L. , Ross, M. S. , & Sah, J. P. (2003). Smoke on the water: the interplay of fire and water flow on Everglades restoration. Frontiers in Ecology and the Environment, 9, 462–468.

[ece38273-bib-0030] Lodge, D. J. , Scatena, F. N. , Asbury, C. E. , & Sanchez, M. J. (1991). Fine litterfall and related nutrient inputs resulting from hurricane Hugo in subtropical wet and lower montane rain forests in Puerto Rico. Biotropica, 23(4a), 336–342.

[ece38273-bib-0031] Lohrey, R. E. , & Kossuth, S. V. (1990). *Pinus elliottii* Engelm. Silvics of North America. Washington: USDA, Forest Service, 1, 338–347.

[ece38273-bib-0032] Loope, L. L. , & Dunevitz, H. L. (1981). Impact of fire exclusion and invasion of *Schinus terebinthifolius* on limestone Rockland pine forests of Southeastern Florida. National Park Service, South Florida Research Center, Everglades National Park.

[ece38273-bib-0033] Loveless, C. M. (1959). A study of the vegetation in the Florida Everglades. Ecology, 40(1), 2–9. 10.2307/1929916

[ece38273-bib-0034] Luviano, N. , Villa‐Galaviz, E. , Boege, K. , Zaldívar‐Riverón, A. , & Del‐Val, E. (2018). Hurricane impacts on plant‐herbivore networks along a successional chronosequence in a tropical dry forest. Forest Ecology and Management, 426, 158–163. 10.1016/j.foreco.2017.09.011

[ece38273-bib-0035] Monnier, Y. , Bousquet‐Melou, A. , Vila, B. , Prévosto, B. , & Fernandez, C. (2013). How nutrient availability influences acclimation to shade of two (pioneer and late‐successional) Mediterranean tree species? European Journal of Forest Restoration, 132, 325–333. 10.1007/s10342-012-0677-7

[ece38273-bib-0036] Noel, J. M. , Maxwell, A. , Platt, W. J. , & Pace, L. (1995). Effects of Hurricane Andrew on cypress (*Taxodium distichum* var. nutans) in south Florida. Journal of Coastal Research, 184–196.

[ece38273-bib-0037] Oberbauer, S. F. , & Strain, B. R. (1984). Photosynthesis and successional status of Costa Rican rain forest trees. Photosynthesis Research, 5(3), 227–232. 10.1007/BF00030022 24458698

[ece38273-bib-0038] Oberbauer, S. F. , & Strain, B. R. (1986). Effects of canopy position and irradiance on the leaf physiology and morphology of *Pentaclethra macroloba* (Mimosaceae). American Journal of Botany, 73, 409–416.

[ece38273-bib-0039] Oberbauer, S. F. , von Kleist, K. , Whelan, K. R. , & Koptur, S. (1996). Effects of Hurricane Andrew on epiphyte communities within cypress domes of Everglades National Park. Ecology, 964–967. 10.2307/2265516

[ece38273-bib-0040] Olmsted, I. C. , Loope, L. L. , & Hilsenbeck, C. E. (1980). Tropical hardwood hammocks of the interior of Everglades National Park and Big Cypress National Preserve. National Park Service, South Florida Research Center, Everglades National Park.

[ece38273-bib-0041] Ostertag, R. , Scatena, F. N. , & Silver, W. L. (2003). Forest floor decomposition following hurricane litter inputs in several Puerto Rican forests. Ecosystems, 6(3), 261–273. 10.1007/PL00021512

[ece38273-bib-0042] Ostertag, R. , Silver, W. L. , & Lugo, A. E. (2005). Factors affecting mortality and resistance to damage following hurricanes in a rehabilitated subtropical moist forest 1. Biotropica, 37(1), 16–24. 10.1111/j.1744-7429.2005.04052.x

[ece38273-bib-0043] Platt, W. J. , Beckage, B. , Doren, R. F. , & Slater, H. H. (2002). Interactions of large‐scale disturbances: Prior fire regimes and hurricane mortality of savanna pines. Ecology, 83(6), 1566–1572.

[ece38273-bib-0044] Platt, W. J. , Doren, R. F. , & Armentano, T. V. (2000). Effects of Hurricane Andrew on stands of slash pine (*Pinus elliottii* var. *densa*) in the everglades region of south Florida (USA). Plant Ecology, 146(1), 43–60.

[ece38273-bib-0045] Poorter, H. , & Nagel, O. (2000). The role of biomass allocation in the growth response of plants to different levels of light, CO_2_, nutrients and water: a quantitative review. Functional Plant Biology, 27(12), 1191.

[ece38273-bib-0046] Popma, J. , & Bongers, F. (1988). The effect of canopy gaps on growth and morphology of seedlings of rain forest species. Oecologia, 75(4), 625–632. 10.1007/BF00776429 28312440

[ece38273-bib-0047] R Core Team (2017). R: A language and environment for statistical computing. R Foundation for Statistical Computing.

[ece38273-bib-0048] Rodriguez‐Garcia, E. , & Bravo, F. (2013). Plasticity in *Pinus pinaster* populations of diverse origins: Comparative seedling responses to light and Nitrogen availability. Forest Ecology and Management, 307, 196–205.

[ece38273-bib-0050] Ruiz, P. L. , Sah, J. P. , Ross, M. S. , Rodriguez, D. L. , & Lambert, A. M. (2011). Monitoring of tree island conditions in the southern Everglades: the effects of hurricanes and hydrology on the status and population dynamics of sixteen tropical hardwood hammock tree islands. Florida International University Digital Commons.

[ece38273-bib-0051] Rybczyk, J. M. , Zhang, X. W. , Day, J. W. Jr. , Hesse, I. , & Feagley, S. (1995). The impact of Hurricane Andrew on tree mortality, litterfall, nutrient influx, and water quality in a Louisiana coastal swamp forest. Journal of Coastal Research, S1(21), 340–353.

[ece38273-bib-0052] Schumacher, E. , Kueffer, C. , Edwards, P. J. , & Dietz, H. (2009). Influence of light and nutrient conditions on seedling growth of native and invasive trees in the Seychelles. Biological Invasions, 11, 1941–1954.

[ece38273-bib-0053] Slater, H. H. , Platt, W. J. , Baker, D. B. , & Johnson, H. A. (1995). Effects of Hurricane Andrew on damage and mortality of trees in subtropical hardwood hammocks of Long Pine Key, Everglades National Park, Florida, USA. Journal of Coastal Research, 197–207.

[ece38273-bib-0054] Stanturf, J. A. , Goodrick, S. L. , & Outcalt, K. W. (2007). Disturbance and coastal forests: A strategic approach to forest management in hurricane impact zones. Forest Ecology and Management, 250(1–2), 119–135.

[ece38273-bib-0055] Tognetti, R. , & Johnson, J. D. (1999). Responses of growth, nitrogen and carbon partitioning to elevated atmospheric CO2 concentration in live oak (*Quercus virginiana* Mill.) seedlings in relation to nutrient supply. Annals of Forest Science, 56(2), 91–105.

[ece38273-bib-0057] van der Sleen, P. , Soliz‐Gamboa, C. C. , Helle, G. , Pons, T. L. , Anten, N. P. R. , & Zuidema, P. A. (2013). Understanding causes of tree growth response to a gap formation: Δ^13^C values in tree rings reveal a predominant effect of light. Trees, 28(2), 439–448.

[ece38273-bib-0058] Visser, J. M. , & Sasser, C. E. (1995). Changes in tree species composition, structure and growth in a bald cypress‐water tupelo swamp forest, 1980–1990. Forest Ecology and Management, 72(2–3), 119–129.

[ece38273-bib-0059] Walters, M. B. , Kruger, E. L. , & Reich, P. B. (1993). Growth, biomass distribution and CO_2_ exchange of northern hardwood seedlings in high and low light: relationships with successional status and shade tolerance. Oecologia, 94(1), 7–16. 28313851 10.1007/BF00317294

[ece38273-bib-0060] Wang, F. , Liu, J. , Zou, B. , Neher, D. A. , Zhu, W. , & Li, Z. (2013). Species‐dependent responses of soil microbial properties to fresh litter inputs in a subtropical forest soil in South China. Journal of Plant Ecology, 8(5), 86–96.

[ece38273-bib-0061] Warren, C. R. , McGrath, J. F. , & Adams, M. A. (2001). Water availability and carbon isotope discrimination in conifers. Oecologia, 127, 476–486. 28547484 10.1007/s004420000609

[ece38273-bib-0062] Whelan, K. R. T. (1997). Short Term Response of Two Cypress Communities Within Everglades National Park To The Effects of Hurricane Andrew. Masters Thesis. Florida International University.

[ece38273-bib-0064] Xi, W. , Peet, R. K. , Lee, M. T. , & Urban, D. L. (2019). Hurricane disturbances, tree diversity, and succession in North Carolina Piedmont forests, USA. Journal of Forestry Research, 30(1), 219–231.

[ece38273-bib-0065] Xu, X. N. , Hirata, E. , & Shibata, H. (2004). Effect of typhoon disturbance on fine litterfall and related nutrient input in a subtropical forest on Okinawa Island., Japan. Basic and Applied Ecology, 5(3), 271–282. 10.1016/j.baae.2004.01.001

[ece38273-bib-0066] Zhang, K. Q. , Simard, M. , Ross, M. , Rivera‐Monroy, V. H. , Houle, P. , Ruiz, P. , Twilley, R. R. , & Whelan, K. R. T. (2008). Airborne laser scanning quantification of disturbances from hurricanes and lightning strikes to mangrove forests in Everglades National Park, USA. Sensors, 8(4), 2262–2292. 10.3390/s8042262 27879821 PMC3673417

[ece38273-bib-0067] Zimmerman, J. K. , Everham, E. M. III. , Waide, R. B. , Lodge, D. J. , Taylor, C. M. , & Brokaw, N. V. (1994). Responses of tree species to hurricane wind in subtropical wet forest in Puerto Rico‐ Implications for tropical tree life histories. Journal of Ecology, 82(4), 911–922.

