# Supplementary material for: Simulated hurricane‐induced changes in light and nutrient regimes change seedling performance in Everglades forest‐dominant species
Source: Ecol Evol. 2021 Dec 14;11(24):17762–73. doi: 10.1002/ece3.8273 (PMC8717270; doi:10.1002/ece3.8273)

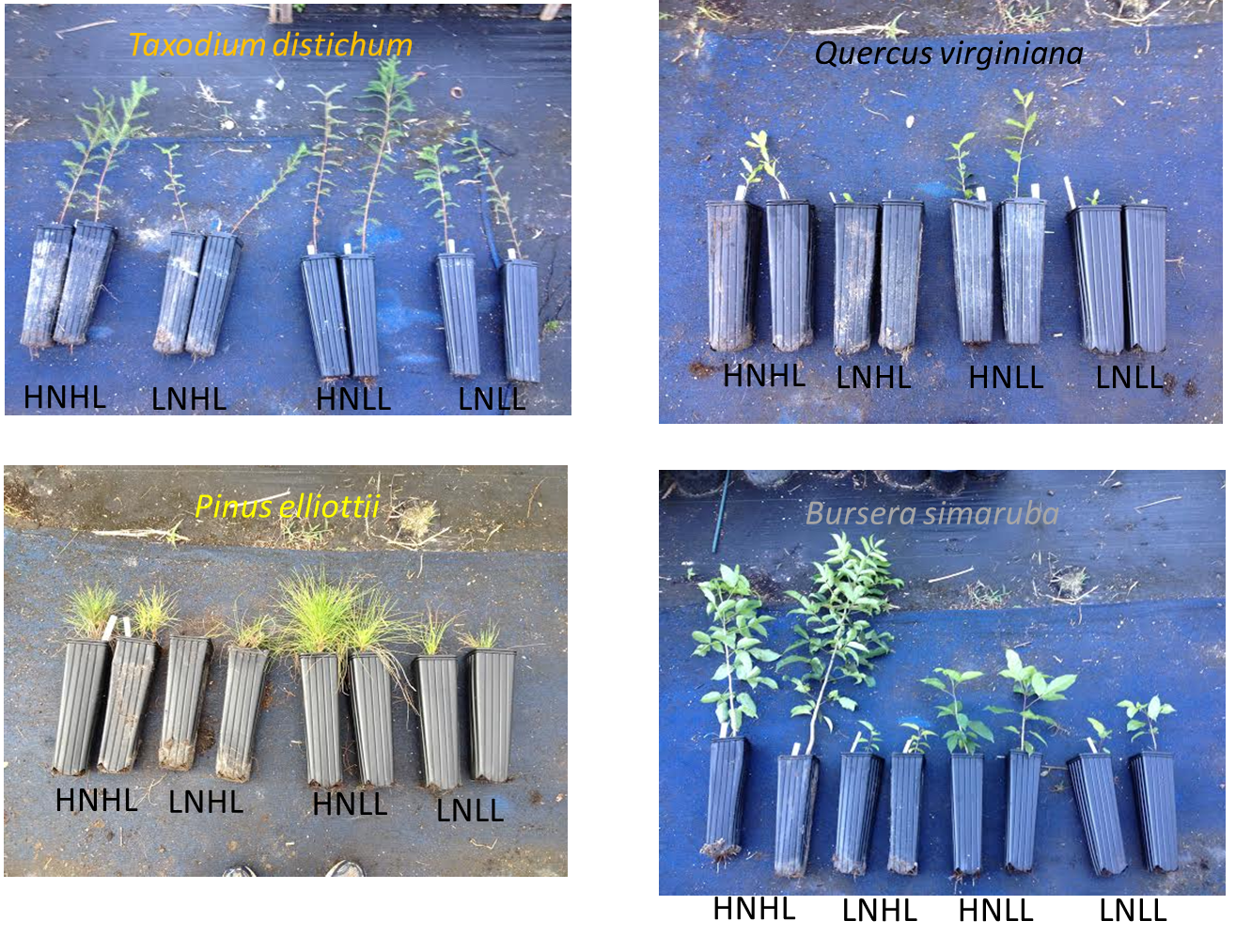


**Figure S1:** Images of the 4 treatments (HNHL, LNHL, HNLL, LNLL) for each study speciess at the conclusion of the study.

**Table S1:** Table of p-values for all treatments and species for two-way ANOVA tests for biomass allocaation measurements and leaf properties, as well as for repeated measure ANOVA tests for weekly photosynthesis and absolute growrh rates. Included are also the p-values for light-nutrient interaction for all measurements and species. Statistically significant p-values are in bold.


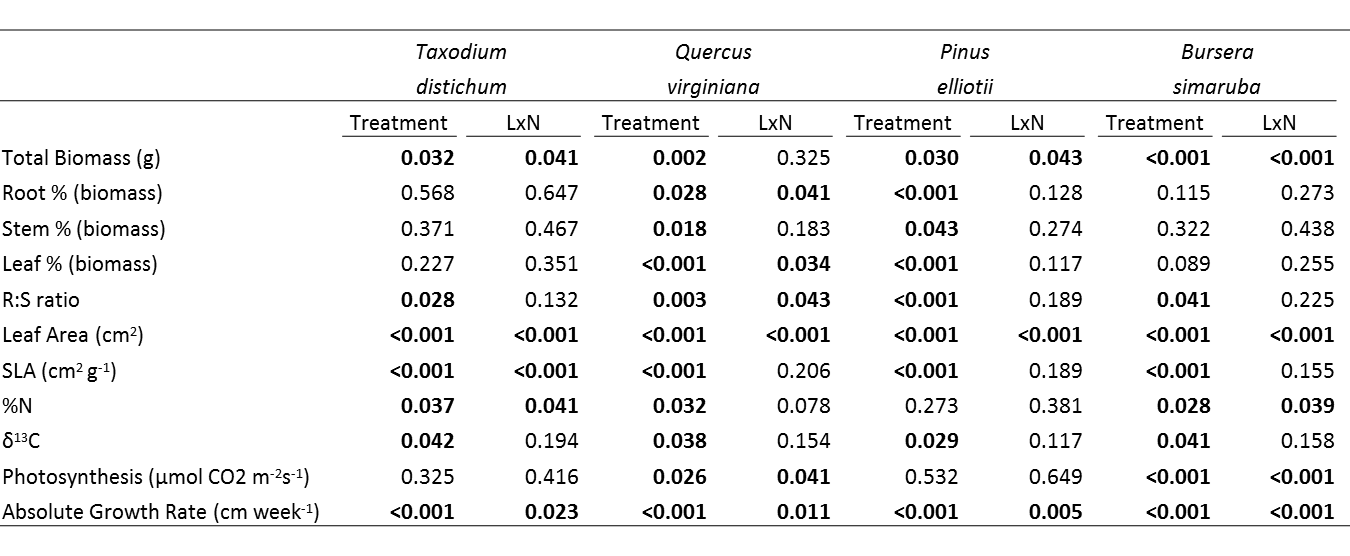

Supplement: Supplementary file 1 — Supplementary Material [file ECE3-11-17762-s001.docx]
